# TREM2 on microglia cell surface binds to and forms functional binary complexes with heparan sulfate modified with 6-O-sulfation and iduronic acid

**DOI:** 10.1016/j.jbc.2024.107691

**Published:** 2024-08-17

**Authors:** Ilayda Ozsan McMillan, Li Liang, Guowei Su, Xuehong Song, Kelly Drago, Hua Yang, Claudia Alvarez, Amika Sood, James Gibson, Robert J. Woods, Chunyu Wang, Jian Liu, Fuming Zhang, Tom J. Brett, Lianchun Wang

**Affiliations:** 1 Department of Molecular Pharmacology and Physiology, University of South Florida Morsani College of Medicine, Tampa, Florida, USA; 2 Departments of Chemistry and Chemical Biology, Center for Biotechnology and Interdisciplinary Studies, Rensselaer Polytechnic Institute, Troy, New York, USA; 3 Department of Biological Sciences, Rensselaer Polytechnic Institute, Troy, New York, USA; 4 Glycan Therapeutics, Raleigh, North Carolina, USA; 5 Complex Carbohydrate Research Center, University of Georgia, Athens, Georgia, USA; 6 Division of Chemical Biology and Medicinal Chemistry, University of North Carolina at Chapel Hill, Eshelman School of Pharmacy, Chapel Hill, North Carolina, USA; 7 Division of Pulmonary and Critical Care Medicine, Department of Internal Medicine, Washington University School of Medicine, St Louis, Missouri, USA

**Keywords:** TREM2, heparan sulfate, binary complex, structure-function, microglia

## Abstract

The triggering receptor expressed on myeloid cells-2 (TREM2), a pivotal innate immune receptor, orchestrates functions such as inflammatory responses, phagocytosis, cell survival, and neuroprotection. TREM2 variants R47H and R62H have been associated with Alzheimer's disease, yet the underlying mechanisms remain elusive. Our previous research established that TREM2 binds to heparan sulfate (HS) and variants R47H and R62H exhibit reduced affinity for HS. Building upon this groundwork, our current study delves into the interplay between TREM2 and HS and its impact on microglial function. We confirm TREM2's binding to cell surface HS and demonstrate that TREM2 interacts with HS, forming HS-TREM2 binary complexes on microglia cell surfaces. Employing various biochemical techniques, including surface plasmon resonance, low molecular weight HS microarray screening, and serial HS mutant cell surface binding assays, we demonstrate TREM2's robust affinity for HS, and the effective binding requires a minimum HS size of approximately 10 saccharide units. Notably, TREM2 selectively binds specific HS structures, with 6-O-sulfation and, to a lesser extent, the iduronic acid residue playing crucial roles. N-sulfation and 2-O-sulfation are dispensable for this interaction. Furthermore, we reveal that 6-O-sulfation is essential for HS-TREM2 ternary complex formation on the microglial cell surface, and HS and its 6-O-sulfation are necessary for TREM2-mediated ApoE3 uptake in microglia. By delineating the interaction between HS and TREM2 on the microglial cell surface and demonstrating its role in facilitating TREM2-mediated ApoE uptake by microglia, our findings provide valuable insights that can inform targeted interventions for modulating microglial functions in Alzheimer's disease.

Triggering receptor expressed on myeloid cells-2 (TREM2), an innate immune receptor, is expressed in various myeloid cells, including dendritic cells, resident macrophages such as osteoclasts and microglia, infiltrating and inflammatory macrophages, and cerebrospinal fluid monocytes. TREM2 is a type-one receptor featuring an extracellular V-type Ig domain, a short stalk, a transmembrane domain that associates with the adaptor protein DAP12 for trafficking and signaling, and a cytoplasmic tail. TREM2 plays a vital role in regulating inflammatory responses, phagocytosis, cell survival, and neuroprotection (1, 2, 3). Identified variants within the TREM2 gene have been implicated as risk factors for multiple neurodegenerative conditions. Specifically, the R47H and R62H variants are associated with Alzheimer's disease (AD), while the T66M variant is linked to Nasu-Hakola disease (4, 5, 6). Although the exact mechanisms by which TREM2 mutations contribute to the disease pathogenesis are not fully understood, accumulating evidence suggests their involvement in dysregulating microglia function, impairing phagocytosis, altering inflammatory responses, and compromising neuronal support and survival (7).

Our previous investigations conducted comprehensive analyses on WT and variant TREM2 proteins, elucidating their structural and functional characteristics (7). Our examination of the TREM2 crystal structure at 3.1 Å revealed distinct patterns: mutations associated with the Nasu-Hakola disease were buried within the protein, while variants linked to AD were located on the protein's surface. This distinction implies that these mutations elicit divergent effects on TREM2 function. By using a combination of biophysical and cellular methodologies, we discerned that Nasu-Hakola mutations significantly impact protein stability, leading to a notable reduction in folded TREM2 surface expression. Conversely, AD risk variants specifically influence the binding affinity of TREM2 to its ligand, heparan sulfate (HS). This observation not only unveils distinct molecular mechanisms underlying the variants associated with Nasu-Hakola and AD pathogenesis but also hints at a potential regulatory role for HS in modulating TREM2 signaling and associated functions in AD (1, 7).

HS is a linear polysaccharide in the glycosaminoglycan family. During biosynthesis, HS covalently binds to protein cores, forming HS proteoglycans. Depending on the protein cores, HS proteoglycans are expressed on cell surfaces and in the extracellular matrix. Their HS moieties interact with many protein ligands, including growth factors, growth factor receptors, morphogens, cytokines, and matrix proteins. These interactions modulate various physiological and pathological processes, such as development, leukocyte trafficking, tumorigenesis, and lipid metabolism (8, 9, 10, 11, 12). Biochemical studies have revealed that HS generally interacts with protein ligands through unique binding sites. These sites consist of relatively small segments of variably sulfated glucosamine and uronic acid epimer residues, including N-, 2-, 6-, and 3-O-sulfation (NS, 2S, 6S, and 3S, correspondingly), as well as epimers of glucuronic acid (GlcA) and iduronic acid (IdoA) arranged in specific patterns (9, 13, 14). In our study, we confirmed that TREM2 binds to the cell surface in an HS-dependent manner and further demonstrated that TREM2 interacts *in cis* with HS, forming TREM2-HS binary complexes on the microglial cell surface. Our biochemical interaction and cell surface binding analyses revealed that TREM2 selectively binds specific HS structures, with 6-O-sulfation and, to a lesser extent, the IdoA residue playing crucial roles. N-sulfation and 2-O-sulfation are dispensable for this interaction. Functional studies further show that HS and 6S facilitate TREM2-mediated ApoE3 uptake in microglia. These findings illuminate the critical role and structure of HS in regulating TREM2 function within microglia, with implications for better understanding neurodegenerative diseases and informing targeted interventions for disease treatment.

## Results

### TREM2 interacts with HS to form TREM2-HS binary complexes on the microglia cell surface

**Figure 1:**
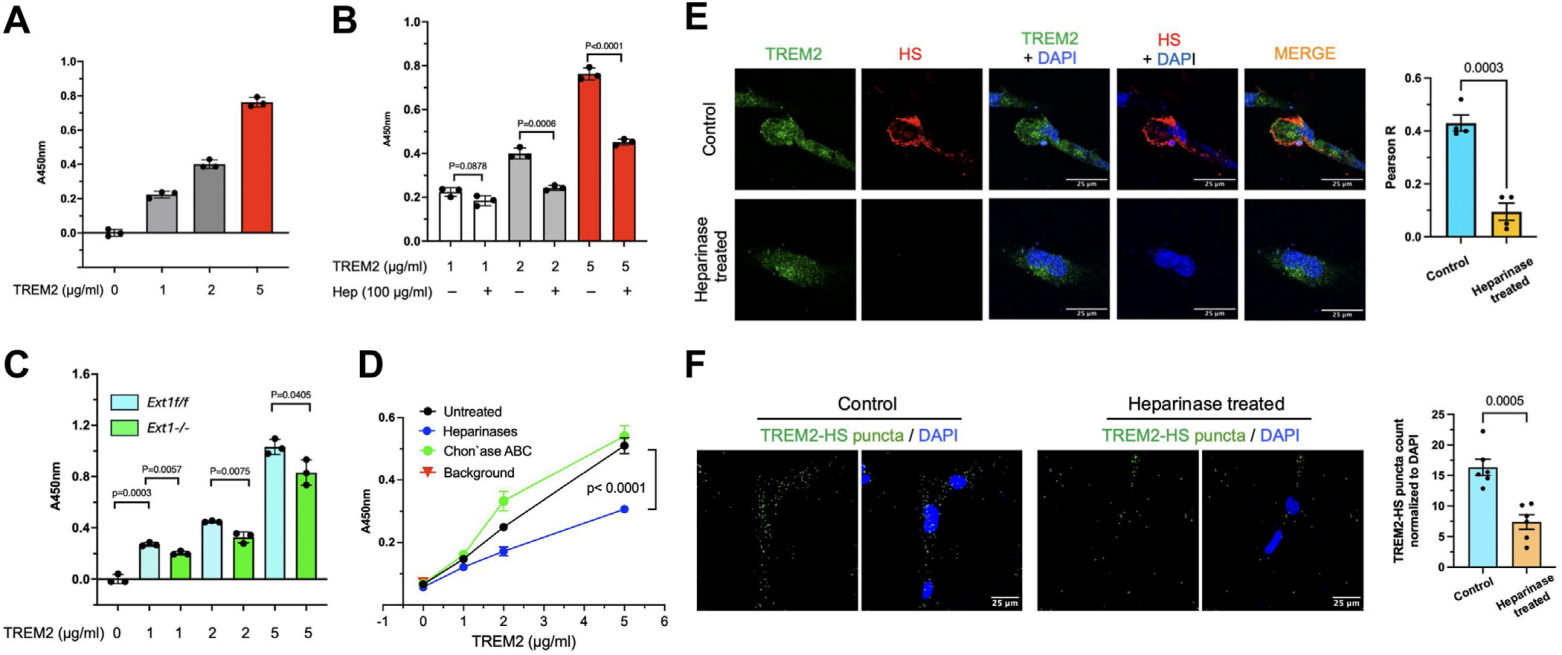
**TREM2 interacts with HS, forming binary complexes on the microglia cell surface.***A*, concentration-dependent binding of TREM2 to WT *Ndst1*^*f/f*^ MLEC surface. A background value of 0.281 (no TREM2 added) was subtracted. *B*, binding of TREM2 to WT *Ndst1*^*f/f*^ MLEC surface in the presence of heparin. *C*, binding of TREM2 to *Ext1*^*−/−*^ mutant MLEC surface. *Ext1*^*−/−*^ MLECs are isogenic mutants derived from *Ext1*^*f/f*^ cells after the conditional deletion of *Ext1* using Adeno-Cre. *D*, binding of TREM2 to WT *Ndst1*^*f/f*^ MLECs after treatment with heparinases I and III or chondroitinase ABC (Chon'ase ABC). The background without streptavidin-HRP addition background is also included. *E*, immunofluorescence staining of TREM2 and HS (using the 10E4 antibody) expressed on the C20 cell surface, with colocalization assessed by Pearson's R correlation analysis. Scale bars represent 25 μm. *F*, proximity ligation assay demonstrating HS-TREM2 binary complexes on the microglial C20 cell surface. The TREM2-HS puncta numbers were calculated by normalizing to nuclear DAPI staining. Data are presented as mean ± SEM and are representative of three independent experiments with statistical significance determined by an unpaired *t* test. Scale bars represent 25 μm. DAPI, 4′,6-diamidino-2-phenylindole; HRP, horseradish peroxidase; HS, heparan sulfate; MLEC, mouse lung endothelial cell; TREM2, triggering receptor expressed on myeloid cells-2.

In a previous study, we examined CHO, THP-1, and N2A cells and observed that the recombinant human and mouse TREM2 ectodomain binds to the cell surface in an HS-dependent manner (7). HS is abundantly expressed on the endothelial cell surface (15), and endothelial cells are commonly used models to determine the HS-depend function of molecules such as growth factors and adhesion molecules (16, 17, 18). In our prior studies, we generated a mouse lung endothelial cell (MLEC) HS mutant library to systemically analyze HS structure-function relationships (15). To determine HS-dependent TREM2 binding on the endothelial cell surface, we tested WT MLEC lines from this library. We examined two WT MLEC lines: one derived from the conditionally targeted HS N-deacetylase/N-sulfotransferases-1 (*Ndst1*^*f/f*^) mice and another from the conditionally targeted exostosin-1 (*Ext1*^*f/f*^) mice. Additionally, we examined an *Ext1*-deficient (*Ext1*^*−/−*^) MLECs derived from *Ext1*^*f/f*^ cells after the targeted *Ext1* alleles were deleted with Adeno-Cre, resulting in deficient HS expression (15). We observed TREM2 concentration-dependent binding to the WT cell surface (Fig. 1*A*). This binding was attenuated by premixing with heparin, a highly sulfated form of HS, which competitively inhibited TREM2 binding to the WT MLEC surface HS (Fig. 1*B* and S1), or by *Ext1* deletion, which abolished HS expression (Fig. 1*C*). In parallel, we treated the cells with heparinases I and III or chondroitinase ABC (chon`ase ABC) and found that heparinases, but not chon`ase ABC, reduced cell surface TREM2 binding (Fig. 1*D*). These data confirm that cell surface TREM2 binding is HS-dependent.

Next, we investigated whether cell surface-expressed TREM2 binds to the cell surface HS using human microglia C20 cells. Immunostaining revealed high expression of both TREM2 and HS on the C20 cell surface (Fig. 1*E*). A significant portion of TREM2 colocalized with HS, quantified by high Pearson`s R correlation coefficient (R = 0.43). This colocalization was attenuated in heparinase-treated cells (R = 0.095). A proximity ligation assay (PLA) was also applied to determine whether TREM2 and HS form binary complexes on the C20 cell surface. After staining with primary rabbit anti-human TREM2 and primary mouse anti-HS 104 antibodies, we incubated the cells with Duolink rabbit plus and mouse minus probes, followed by ligation and amplification steps. We observed abundant TREM2-HS binary complexes, indicated by red dots on the C20 cell surface (Fig. 1*F*). Notably, TREM2-HS complex formation was dramatically reduced when the cell surface HS was degraded by heparinase treatment before PLA staining. Our colocalization and PLA results demonstrate that TREM2 interacts with HS *in cis* to form TREM2-HS binary complexes on the microglial cell surface, suggesting that HS may modulate TREM2 signaling and microglia functions.

### TREM2 exhibits high-affinity binding to heparin which is dependent on the size of the HS oligosaccharide

**Figure 2:**
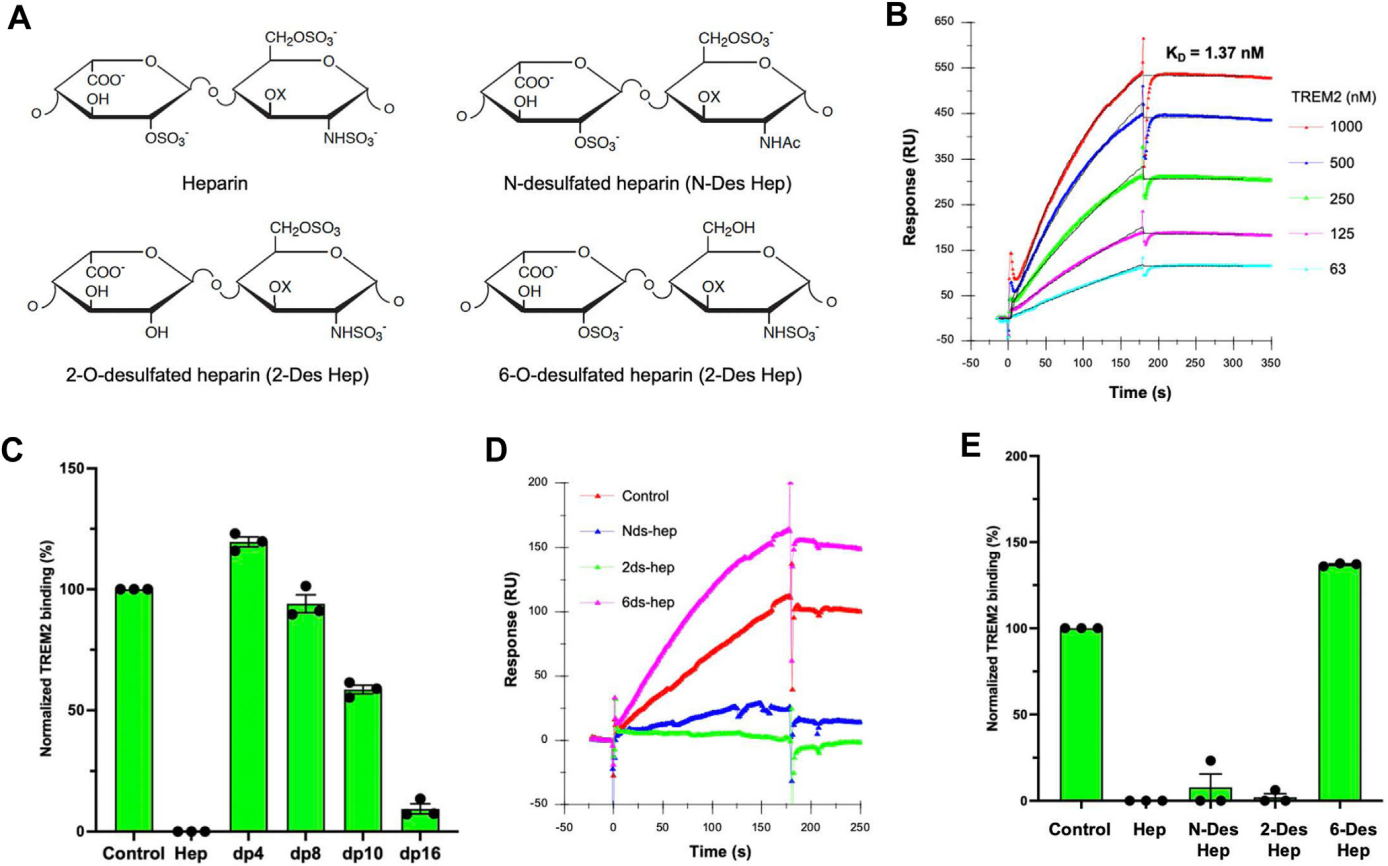
**Interaction of TREM2 with heparins of different sizes and chemical modifications.***A*, depiction of the structures of chemically modified heparins. *B*, the SPR binding sensorgram demonstrates strong TREM2 binding affinity to immobilized heparin, with a KD calculated at 1.37 ± 1.28 × 10ˆ-9M. *C*–*E*, competitive SPR assays involved preincubating TREM2 (250 nM) with 1000 nM heparin oligomers of varying sizes (*C*) or with 1000 nM heparin, N-Desulfated Heparin (N-Des Hep), 2-Desulfated Heparin (2-Des Hep), or 6-Desulfated Heparin (6-Des Hep) (*D* and *E*). These results are representative of three independent experiments. SPR, surface plasmon resonance; TREM2, triggering receptor expressed on myeloid cells-2.

In our previous investigation, we identified a conserved surface site on TREM2 that contains residues associated with AD risk variants (R47H and R62H) and a protective variant (T96K) (7). This site also binds to HS, with R47H and R62H mutations decreasing HS binding, while T96K mutation enhances HS binding (7). These findings underscore the critical role of altered TREM2-HS interaction in modulating AD risk. However, the TREM2 binding affinity and the specific structure of HS molecule involved in this interaction remain unknown. To investigate TREM2-HS interaction in greater detail, we used heparin, which is a highly sulfated analog of HS commonly used as a surrogate. In surface plasmon resonance (SPR) experiments, we immobilized biotinylated streptavidin sensor chips and generated sensorgrams depicting the interaction between TREM2 and chip surface-immobilized heparin by injecting TREM2 at various concentrations (1000, 500, 250, 125, and 63 nM) (Fig. 2, *A* and *B*). The sensorgrams obtained at different TREM2 concentrations were globally fitted to the 1:1 Langmuir binding model, revealing a dissociation constant (K_D_) of 1.37 ± 1.20 × 10^−9^ M, that indicated a very high binding affinity between TREM2 and heparin. Furthermore, we conducted solution/surface competitive TREM2 binding SPR analysis to determine the minimum heparin size required to bind to TREM2. Heparin-derived oligosaccharides of various sizes, ranging from dp4 to dp16, were examined at 1000 nM in the solution phase and premixed with TREM2 at 250 nM. Heparin completely inhibited TREM2 binding to surface-immobilized heparin (Fig. 2*C*). While oligosaccharides dp4 and dp8 showed no competition, significant competition was observed for the dp10 oligosaccharide (45% inhibition), and dp16 exhibited potent inhibition (93%). These findings indicate that the binding of HS to TREM2 requires a minimum heparin size of approximately ten saccharide units.

### 6-O-sulfation and, to a lesser extent, iduronic acid, but not N- and 2-O-sulfation, are required for HS to bind TREM2

HS contains common sulfation modifications important for binding protein, including N-sulfation (NS), 2-O-sulfation (2S), and 6-O-sulfation (6S), along with the less common 3-O-sulfation (3S) (16, 19, 20). To pinpoint the essential sulfation modification for HS binding to TREM2, we used an SPR competition assay in the presence of native heparin or chemically modified heparin variants, including N-desulfated heparin (N-Des Hep), 2-O-desulfated heparin (2-Des Hep), and 6-O-desulfated heparin (6-Des Hep). While heparin, N-Des Hep, and 2-Des Hep displayed potent and similar inhibitory activities, 6-Des Hep showed none, indicating the necessity of 6S for HS binding to TREM2 (Fig. 2, *D* and *E*).

**Figure 3:**
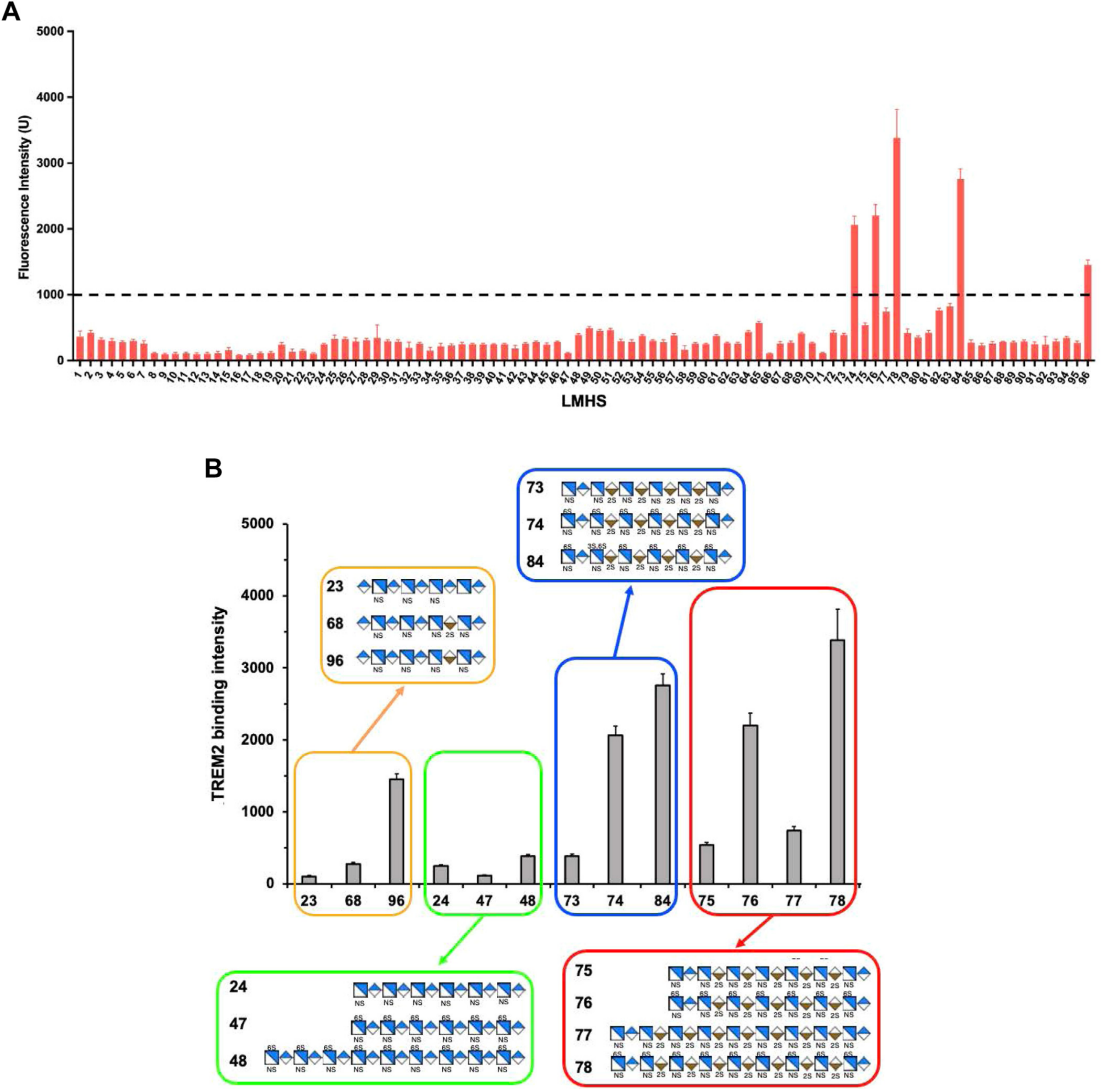
**Low molecular weight HS microarray reveals TREM2 binds to HS structures containing 6-O-sulfates and iduronic acid modifications.***A*, the fluorescence intensity of bound TREM2 to wells immobilized with chemoenzymatically synthesized low molecular weight HS with different structures. TREM2 with his-tag (10 μg/ml) was screened against 96 LMW HS compounds printed at 25 μM, and bound TREM2 was visualized and quantified using AF488 NTA Ni. Each compound was printed 12 times, resulting in 12 spots per compound. The binding threshold for this experiment was set at 1000 fluorescence intensity (U), depicted by a *black dashed line*. Results are represented as mean ± SD for each compound. *B*, comparison of TREM2 binding HS oligosaccharides (HS # 74, 76, 78, 84, and 96) and compounds that do not bind TREM2 (HS#23, 24, 47, 48, 68, 73, 75, and 77). HS, heparan sulfate; LMW, low molecular weight; TREM2, triggering receptor expressed on myeloid cells-2.

Considering the structural heterogeneity of heparin and its derivatives, we used a low molecular weight (LMW) HS microarray to elucidate the specific HS structure needed for TREM2 binding. This microarray facilitates the simultaneous investigation of protein-HS interactions across various HS structures with defined chain lengths, sequence, and sulfation patterns (Fig. S2) (21, 22, 23). Antithrombin binding to known HS structures within the microarray served as an analysis quality control (Fig. S3). Among the 96 HS oligosaccharides probed, only five exhibited binding to TREM2, including HS oligosaccharides #74, #76, #78, #84, and #96 (Figs. 3*A* and S4, and Table S1). Among those, #78 was the strongest binder and had much better binding to TREM2 than #77. #78 differs from #77 only by additional 6S modifications (Fig. 3*B*) (Table S1), affirming a clear dependence on 6S for TREM2-HS interaction observed by SPR competition analysis. Similar trends for 6S dependence were observed in the comparison of #84 *versus* #73 and #76 *versus* #75. The TREM2-bound HS oligosaccharides ranged in size from 9 to 18 saccharide residues, affirming a minimum requirement of approximately ten monosaccharide units for TREM2 binding (Fig. 3*B*).

Intriguingly, the presence of a 3S modification in HS#84, compared to HS#74, significantly enhanced TREM2 binding, suggesting a contributory role of 3S. This is reminiscent of 3S dependence of HS binding of other proteins associated with AD, such as tau and ApoE (22, 23, 24, 25).

HS#23, #68, and #96 are all 9-mers, yet only HS#96 exhibited weak but significant binding to TREM2, whereas the other two did not bind. Comparing HS#23 to HS#96 reveals a substitution of GlcA with IdoA, suggesting a role of IdoA in TREM2 binding. However, in HS#68, an additional 2S modification on the IdoA, compared to HS#96, implies a blocking effect on TREM2 binding. Interestingly, TREM2-binding HS oligomers, such as #74, 76, 78, and 84, contain IdoA-2S residues, suggesting that the presence of 6S in longer HS chains may overcome the inhibitory effect of 2S.

Moreover, other HS oligomers that are larger and have higher overall sulfation levels, such as HS#24, #47, and #48 (ranging from 12 to 18 mers with 6–18 sulfates), did not bind TREM2. This contrasts with observations for HS#96, a 9-mer with only four sulfates, and HS#74, a 12-mer with 16 sulfates, both exhibited binding to TREM2. Therefore, the interaction between HS and TREM2 is not contingent upon the overall sulfation level but rather necessitates a specific HS structural motif.

In summary, our SPR analyses involving chemically modified heparins, alongside LMW HS microarray analysis, indicate that TREM2 binds selectively to a particular HS structure, with 6S being indispensable for HS-TREM2 binding. Additionally, our findings suggest the contributory roles of other modifications in the binding, including IdoA residue and 3S.

### Deficiencies of *Hs6st*, *Ndst1*, and *Glce*, but not *Hs2st* and *Hs3st1*, attenuate cell surface HS binding of TREM2

**Figure 4:**
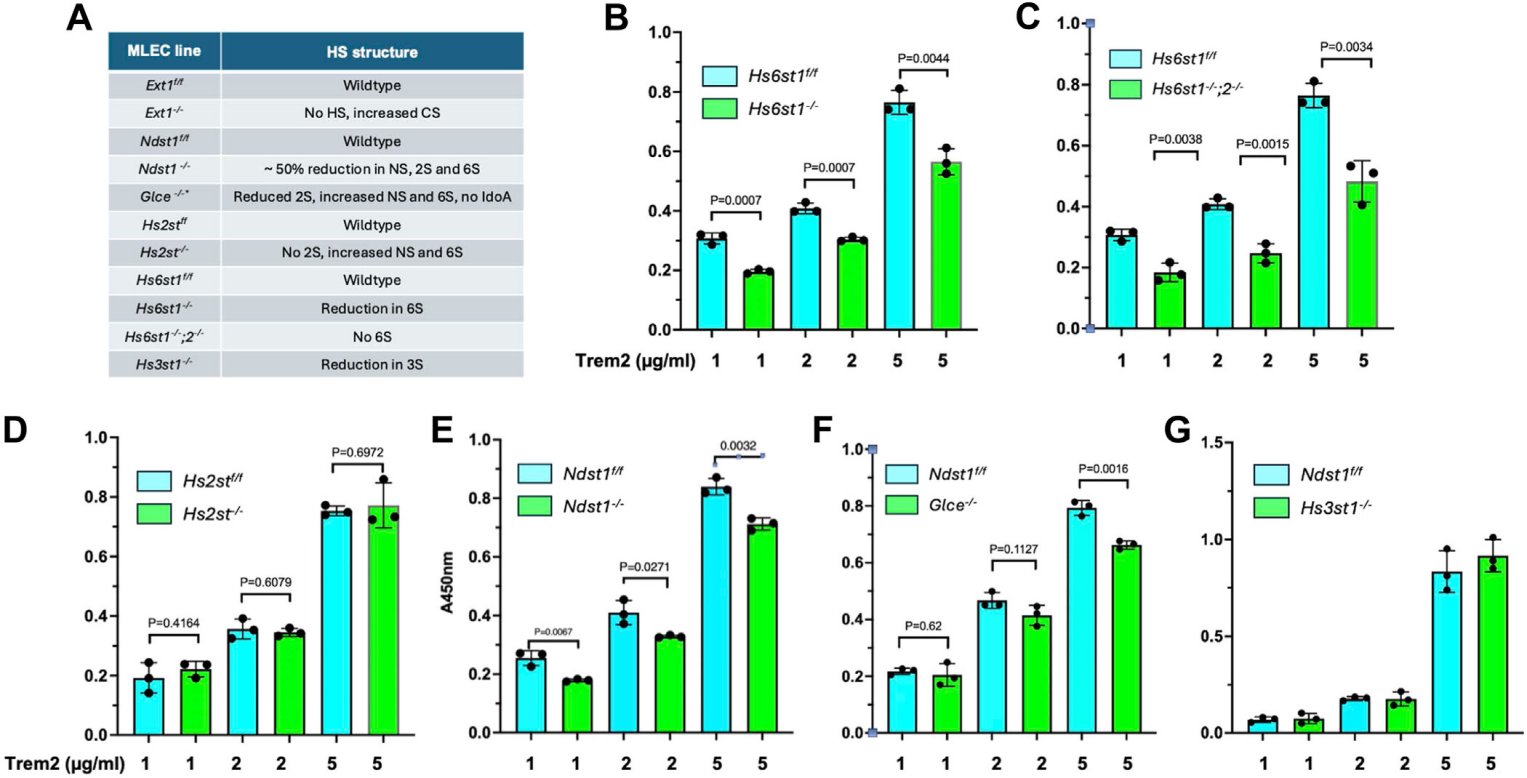
**Binding of TREM2 to HS mutant MLEC cell surfaces.***A*, generated HS mutant MLEC lines, which express differently structured HS. *B*, comparison between *Hs6st1*^*−/−*^*versus* corresponding isogenic WT *Hs6st1*^*f/f*^ MLECs. *C*, comparison between *Hs6st1*^*−/−;*^*2*^*−/−*^*versus* corresponding *isogenic* WT *Hs6st1*^*f/f*^ MLECs. *D*, comparison between *Hs2st*^*−/−*^*versus* corresponding isogenic WT *Hs2st*^*f/f*^ MLECs. *E*, comparison between *Ndst1*^*−/−*^*versus* corresponding isogenic WT *Ndst1*^*f/f*^ MLECs. *F*, comparison between *Glce*^*−/−*^*versus* corresponding isogenic WT *Ndst1*^*f/f*^ MLECs. *G*, comparison between *Hs3st1*^*−/−*^*versus* corresponding isogenic WT *Ndst1*^*f/f*^ MLECs. The data presented are representative of three independent experiments and are expressed as mean ± SEM. An unpaired *t* test was applied for statistical analysis. Glce, glucuronyl C5-epimerase; HS, heparan sulfate; Hs2st, HS 2-O-sulfotransferase; MLEC, mouse lung endothelial cell; TREM2, triggering receptor expressed on myeloid cells-2.

The biosynthesis of HS occurs within the Golgi apparatus and involves a series of enzymes from multiple families [26, 27]. The heterodimer Ext1/2 acts as a copolymerase, elongating the HS chain by sequentially adding GlcA and N-acetylglucosamine (GlcNAc) residues. As the HS chain grows, it undergoes various modifications, including NS of GlcNAc by Ndst1-4, conversion of GlcA to IdoA by glucuronyl C5-epimerase (Glce) and, 2S of IdoA, and less frequently of GlcA, by HS 2-O-sulfotransferase (Hs2st), as well as 6S and 3S of N-sulfated GlcNAc (GlcNS) by HS 6-O-sulfotransferase1-3 (Hs6st1-3) and HS 3-O-sulfotransferase1-6 (Hs3st1-6), respectively. Once synthesized, HS may undergo further modifications on the cell surface and in the extracellular matrix by 6-O-endosulfatases (Sulf1-2) enzymes, which remove 6S from GlcNS [28–32]. The abundance and structure of HS expression are intricately regulated by the concerted actions of the enzymes responsible for its biosynthesis and remodeling. In our previous study, we established a comprehensive HS mutant MLEC library using Cre-LoxP gene targeting and CRISPR-Cas9 approaches (15). This library enabled us to precisely assess the contribution of various HS modifications to TREM2 binding in a cellular context (15). To elucidate the specific roles of NS, 2S, 6S, 3S, and epimerization in HS binding to TREM2, we systematically analyzed the cell surface binding of TREM2 to HS mutant cells, including *Ndst1*^*−/−*^, *Hs2st*^*−/−*^, *Hs6st1*^*−/−*^, *Hs6st1*^*−/−*^;*2*^*−/−*^, *Hs3st1*^*−/−*^, and *Glce*^*−/−*^ MLECs, along with their corresponding isogenic WT controls (Fig. 4*A*). We incubated cells with TREM2 at concentrations of 1, 2, and 5 μg/ml, and observed reduced cell surface TREM2 binding on the *Hs6st1*^*−/−*^, and *Hs6st1*^*−/−*^*;2*^*−/−*^ cell surface across all examined TREM2 concentrations (Fig. 4, *B* and *C*). Since *Hs6st1*^*−/−*^, and *Hs6st1*^*−/−*^*;2*^*−/−*^ cells partially or completely lack 6S, respectively, the diminished cell surface binding reiterates that 6S is essential for cell surface HS binding to TREM2. Conversely, *Hs2st*^*−/−*^ cells, which lack 2S and have compensatory increases in NS and 6S, did not show altered TREM2 binding (Fig. 4*D*), highlighting the dispensable nature of 2S in this interaction. Furthermore, we observed reduced TREM2 binding on *Ndst1*^*−/−*^ cell surfaces (Fig. 4*E*), likely due to decreased 6S content in *Ndst1*^*−/−*^ HS, since N-desulfation did not affect heparin binding to TREM2, and 2S is dispensable for cellular HS to bind TREM2. The *Glce*^*−/−*^ and *Hs3st1*^*−/−*^ MLECs were generated from the WT *Ndst1*^*f/f*^ cells through CRISPR-Cas9-mediated gene deletion (15). TREM2 at a concentration of 5 μg/ml showed reduced cell surface binding on *Glce*^*−/−*^ cells compared to their isogenic *Ndst1*^*f/f*^ counterparts (Fig. 4*F*). *Glce*^*−/−*^ HS exhibits decreased 2S and increased NS and 6S, and lacking IdoA compared to its isogenic WT *Ndst1*^*f/f*^ HS. These structural features suggest that the reduced TREM2 binding is due to the deficiency of IdoA, considering that NS and 2S did not affect TREM2 binding even though 6S was increased. *Hs3st1*^*−/−*^ MLECs exhibited unchanged cell surface TREM2 binding compared to their isogenic *Ndst1*^*f/f*^ counterparts (Fig. 4*G*). Since *Hs3st1* deletion only partially reduces 3S (15) and different Hs3sts may generate distinct 3S-modified HS structures (26), the contribution of 3S produced by other Hs3sts to TREM2 binding cannot be excluded. Overall, our serial HS mutant cell studies revealed that 6S and, to a lesser extent, IdoA are required for HS to bind TREM2 on the cell surface, while NS and 2S play nonessential roles in this process.

### Deficiencies in *Hs6sts* disrupt microglia cell surface TREM2-HS binary complex formation

**Figure 5:**
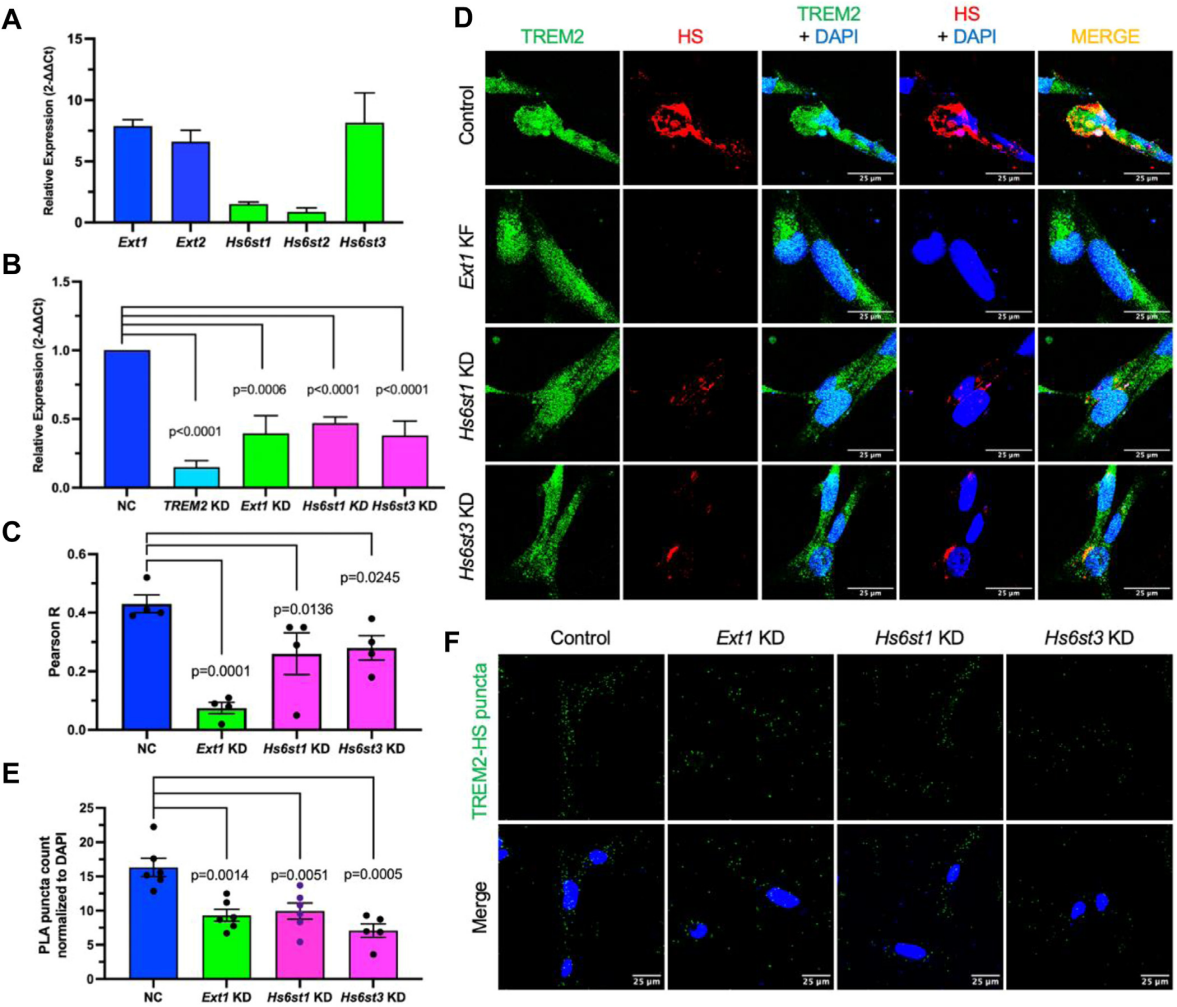
***Ext1* and *Hs6sts* are required for TREM2-HS binary complex formation on the microglial cell surface.***A*, expression profiles of *Ext1*, *Ext2*, and *Hs6st1-3* in C20 microglia cells assessed by quantitative RT-PCR analysis. *B*, siRNA knockdown (KD) of *TREM2*, *Ext1*, *Hs6st1*, and *Hs6st3* in C20 cells compared to empty vector-transfected cells (normal control, NC). *C* and *D*, impact of *Ext1*, *Hs6st1*, or *Hs6st3* knockdown on the colocalization of TREM2 and HS on C20 cell surface. The images in Figures 1*E* and 5, *C* and *D* were acquired from the same set of experiment and the control is reused here. *E* and *F*, effect of *Ext1*, *Hs6st1*, or *Hs6st3* knockdown on TREM2-HS binary complex formation on C20 cell surface. The images in Figures 1*E* and 5, *E* and *F* were acquired from the same set of experiment and the control is reused here. Data are presented as mean ± SEM and are representative of three independent experiments. Scale bars represent 25 μm. HS, heparan sulfate; Hs6st1-3, HS 6-O-sulfotransferase1-3; TREM2, triggering receptor expressed on myeloid cells-2.

To determine the requirement of 6S for HS binding to microglial cell surface-expressed TREM2, we initially profiled HS gene expression in C20 cells. C20 cells express *Ext1* and *Ext2* (Fig. 5*A*). Among the *Hs6st* family, C20 cells exhibit high expression of *Hs6st3*, a moderate expression of *Hs6st1*, and a low expression of *Hs6st2* (Fig. 5*A*). We knocked down *Ext1*, *Hs6st1*, and *Hs6st3* using specific siRNAs (Fig. 5*B*). Immunofluorescence staining revealed high expression levels of TREM2 and HS on the cell surface, with significant colocalization between them (Pearson R value = 0.420) (Fig. 5, *C* and *D*). The HS expression and TREM2 colocalization were diminished in *Ext1* knockdown C20 cells, validating the efficiency of the analysis. Notably, the colocalization of HS and TREM2 showed a decreasing trend in C20 cells with individual knockdowns of either *Hs6st1* or *Hs6st3*. Additionally, PLA using anti-HS 10E4 and anti-human TREM2 antibodies revealed abundant TREM2-HS binary complex puncta in empty vector-transfected C20 cells (Fig. 5, *E* and *F*). Importantly, the number of these puncta significantly decreased upon individual knockdowns of *Ext1*, *Hs6st1*, or *Hs6st3*. These findings confirm that HS binds TREM2 *in cis* on the microglia cell surface and highlight the essential role of HS-6S in this binding and binary complex formation process.

### Deficiencies in *Ext1* and *Hs6st* disrupt microglial uptake of ApoE3 protein

**Figure 6:**
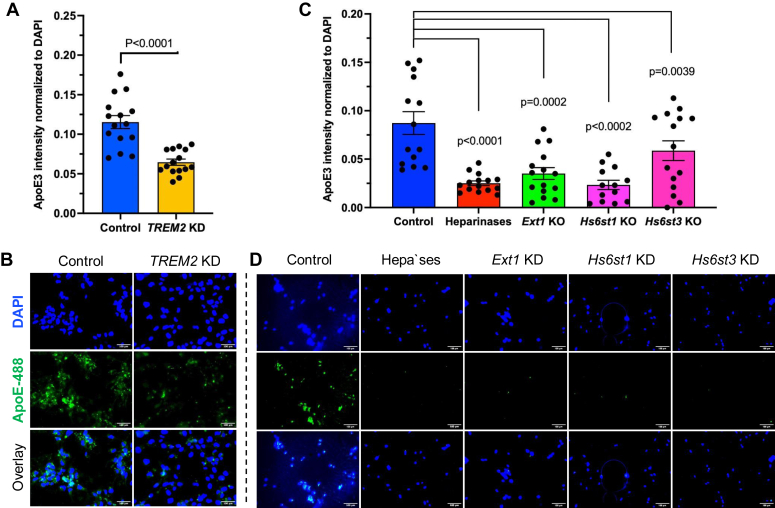
**Impact of *TREM2*, *Ext1*, *Hs6st1*, and *Hs3st6* on ApoE uptake by C20 microglia.***A* and *B*, *TREM2* knockdown in C20 cells results in decreased uptake of ApoE-488 compared to cells transfected with an empty vector. *C* and *D*, knockdown of *Ext1*, *Hs6st1*, or *Hs3st3* in C20 cells leads to reduced uptake of ApoE-488 compared to cells transfected with an empty vector. Scale bars represent 100 μm. Data are presented as mean ± SEM and are representative of three independent experiments. TREM2, triggering receptor expressed on myeloid cells-2; Hs6st1, HS 6-O-sulfotransferase1.

ApoE is a 299-amino acid protein that serves as a major cholesterol carrier in circulation and the sole cholesterol transporter in the brain (27). Genome-wide association studies (GWAS) and whole-exome sequencing have identified over 30 risk loci associated with AD (28), including ApoE and TREM2 (29, 30). TREM2 has been demonstrated to bind to ApoE and act as its receptor (31, 32, 33), suggesting that the ApoE-TREM2 interaction may modulate AD pathogenesis. TREM2 mediates ApoE uptake in microglia (30). We confirmed this by demonstrating that *TREM2* knockdown attenuated the uptake of human ApoE3 conjugated with Alexa Fluor 488 (ApoE3-488) (34, 35) by C20 cells (Figs. 5*B* and 6, *A* and *B*). Furthermore, ApoE3-488 uptake by C20 cells was significantly reduced upon individual knockdown of *Ext1*, *Hs6st1*, or *Hs6st3*, indicating that HS and HS-6S facilitate TREM2-mediated ApoE3 uptake in microglia (Fig. 6, *C* and *D*). Previous studies, including ours (23, 34, 35), have demonstrated that ApoE3 binds to HS. Therefore, our new findings suggest that HS may act as a coreceptor, simultaneously interacting with ApoE3 and TREM2 on the microglial cell surface to facilitate ApoE3 uptake.

## Discussion

Recent studies on human populations have highlighted the critical role of HS in AD. The loss-of-HS binding ApoE3 Christchurch variant (R136S) confers protective effects in autosomal dominant AD patients with PSEN1 mutations (36, 37) and in AD mouse models (38, 39). Conversely, the RELN variant (H3447R), characterized by heightened HS binding, also provides protection in autosomal dominant AD patients and mouse models (40). These protective effects associated with the APOE3 Christchurch variant and the RELN (H3447R) variant are believed to arise from alterations in HS binding (36, 37, 38, 39, 40). Meanwhile, recent studies have emphasized the importance of TREM2 and neuroinflammation in neurodegenerative diseases such as AD and Nasu-Hakola disease (2, 41, 42, 43, 44). Understanding the impact of disease-associated genetic mutations in TREM2 on its function is crucial for developing targeted therapies. Our earlier studies demonstrated that AD-risk variants R47H and R62H disrupt TREM2's interaction with HS, suggesting a role for HS in TREM2 function and AD progression (7). Our current study further verifies HS as a TREM2 ligand and reveals the formation of TREM2-HS complexes on microglial surfaces. Moreover, our functional investigations demonstrate that HS facilitates TREM2-mediated uptake of ApoE3 by microglia. These findings underscore the regulatory role of HS in TREM2 signaling and microglial functions, offering valuable insights for future research and potential therapeutic strategies.

Understanding the specific HS structures necessary for binding to TREM2 is crucial for developing targeted therapies. In our LMW HS microarray analysis, we observed significant variation in TREM2 binding among HS oligomers with similar sizes and sulfation levels. This indicates that the overall sulfation level alone does not solely determine HS binding to TREM2. Rather, specific HS structures play a critical role in this interaction. Through comparisons of TREM2-binding and nonbinding HS oligomers with similar sequences, SPR competition analysis of chemically modified heparins, and examination of HS modification-deficient cells, we identified essential HS structural requirements for TREM2 binding. These include 6S and a minimum chain length of approximately 10 saccharides, with additional contributions from IdoA and possibly 3S, while NS and 2S are dispensable. Determining the precise arrangement of these modifications within the specific TREM2-binding motif will be crucial for future studies. This knowledge holds significant therapeutic potential, guiding the development of targeted treatments to modulate TREM2 function in neurodegenerative disease management.

To gain deeper insights into the TREM2-HS interaction, including the specific TREM2 amino acid residues involved, our current study also created an *in silico* model of the interaction between a heparin octa-saccharide fragment ([IdoA2S-GlcNS6S]_4_) and the A chain of TREM2 (Methods and Results in the Supplemental Information). Focused docking resulted in 20 ligand poses. The top-ranked pose (Fig. S5), based on theoretical interaction energy, was used for visualization and further analysis. According to protein-ligand interaction profiler analysis, the heparin fragment formed hydrogen bonds and/or salt bridge interactions with seven residues of the TREM2 protein, including four arginines (R47, R62, R76, and R77), one asparagine (N68), one serine (S65), and one tryptophan (W70) (Table S2). The values of the glycosidic torsion angles were all within allowable regions of the potential energy surface (45, 46). The modeling indicated that the octamer-fragment of heparin could occupy the entire putative binding site in TREM2. The experimental observation that binding to heparin oligomers was enhanced as the heparin chain length increases may arise from mass transfer (or ligand rebinding) effects due to the increase in the number of binding epitopes or may indicate an alternative binding mode. Further modeling and/or experimental studies are required to quantify the roles of each modification moiety of HS and the amino acid residues of TREM2. Nevertheless, our present model provides a basis for the design of such studies.

AD is a progressive neurodegenerative disorder characterized by the accumulation of amyloid-β (Aβ) plaques, intraneuronal neurofibrillary tangles, and widespread neuroinflammation, including microglial activation. Despite extensive research, the precise molecular mechanisms underlying AD pathogenesis remain elusive. GWAS and whole-exome sequencing have identified over 30 AD risk loci, including TREM2 and ApoE (28, 29, 30). TREM2, a cell surface receptor on myeloid cells, initiates multiple pathways upon ligand binding, promoting cell survival, proliferation, chemotaxis, and phagocytosis, essential for normal immune function (47). Notably, TREM2 is highly expressed in microglia, the resident immune cells of the central nervous system. The loss-of-function R47H mutation in TREM2 represents one of the strongest single-allele genetic risk factors for AD (7, 47), implicating microglial dysfunction in AD pathogenesis. APOE, a major cholesterol carrier in circulation and the sole cholesterol transporter in the brain, has three isoforms: AD-protective ApoE2, neutral ApoE3, and high AD-risk ApoE4 (48). TREM2 binds all three ApoE isoforms with high affinity, mediating phagocytosis of ApoE-bound apoptotic neuronal cells or Aβ by microglia or macrophages (31, 32, 33). Interestingly, the R47H mutation impairs TREM2’s ability to bind both HS and ApoE (31, 32, 33). Previous studies, including our own, have demonstrated that HS also binds ApoE (23, 34, 35), suggesting that HS may simultaneously interact with TREM2 and ApoE to facilitate TREM2-mediated ApoE uptake by microglia. Our current study supports this by demonstrating that the loss of HS disrupts TREM2-mediated ApoE uptake by microglia. We propose a model in which cell surface HS acts as a coreceptor for ApoE, bridging the formation of a functional ApoE/HS/TREM2 ternary complex during microglial ApoE uptake. This process is disrupted by the AD-associated R47H mutation, contributing to AD pathogenesis. Additionally, TREM2 recognizes other ligands such as phospholipids, glycolipids, and lipoproteins like low-density lipoprotein and high-density lipoproteins, as well as clusterin (31, 32, 33). Investigating whether HS regulates TREM2 interactions with these ligands and related biological processes presents an intriguing avenue for future research.

Several investigations have reported abnormal HS expression in the AD brain, including elevated HS levels (49, 50, 51, 52) and structural alterations evidenced by changes in protein-ligand interactions (50) and direct biochemical analyses (24, 53). Gene expression profiling has revealed dysregulation of HS biosynthesis genes, notably upregulation of *Hs6st1* and *Hs6st2* (54), enzymes that add 6-O-sulfate groups to HS, and downregulation of *Sulf2* (55, 56), which removes 6-O-sulfated groups. This dysregulation may enhance the HS-TREM2 interaction within microglia *in cis* or between different cell types *in trans*, potentially influencing AD pathogenesis. Similarly, upregulated expression of *Glce* and *Hs3sts* in AD (54, 56), enzymes responsible for converting GlcA to IdoA and adding 3-O-sulfate groups to HS, respectively, suggest a potential role for elevated IdoA and 3S in enhancing TREM2-HS interaction and contributing to AD pathogenesis. Notably, the level of 3-O-sulfated HS is increased in the AD brain (24, 53), and 3S enhances the binding of HS to tau (22, 24) and ApoE (23). Consistent with these findings, our glycan microarray data indicate that HS binding to TREM2 may be enhanced by 3S, supporting a role for 3S in AD. This could offer a potential mechanistic link underlying the genetic association between *Hst3st1* and AD identified in GWAS (57, 58, 59).

In summary, our research has elucidated the molecular intricacies of the interaction between TREM2 and HS, forming functional TREM2-HS complexes on microglia cell surfaces. This highlights the role of HS in modulating microglial functions through its interaction with TREM2. Importantly, our investigations have identified the specific HS structure to which TREM2 binds, revealing crucial HS modifications necessary for this interaction. These findings carry significant implications, particularly in developing therapeutics targeting neuroinflammation. By selectively targeting the TREM2 pathway based on its interaction with specific HS structures, we may unlock novel treatment avenues for AD and other neurodegenerative diseases. Furthermore, understanding the potential impact of aberrant HS expression on the TREM pathway in AD patients may offer invaluable insights into disease mechanisms, potentially paving the way for developing biomarkers and precision medicine strategies for AD.

## Experimental procedures

### Cell surface TREM2 binding assay

The biotinylated recombinant human TREM2 extracellular domain was produced as previously described (7) and used to assess its cell surface binding. Initially, MLEC lines, which were generated in our lab and comprised both WT and multiple HS mutants (15, 60, 61), were plated at a density of 4 × 10^4^ cells per well in a 96-well plate containing Dulbecco’s modified Eagle medium (DMEM) supplemented with 10% fetal bovine serum (FBS), 100 U/ml penicillin, and 100 μg/ml streptomycin. After an overnight culture at 37 °C with 5% CO_2_, the cells were fixed using 4% paraformaldehyde in PBS at room temperature (RT) for 15 min. After washing and overnight blocking in 5% bovine serum albumin at 4 °C, the cells were incubated with biotinylated TREM2 at 0, 1, 2, or 5 μg/ml for 1 h at RT. Following another round of washing, the cells were incubated with streptavidin-horseradish peroxidase (1:5000 dilution) for 1 h at RT. After thorough washing, a horseradish peroxidase substrate was added, and the reaction was allowed to proceed for 25 min before terminating with 0.5 M HCl. Absorbance at 450 nm was then measured. We confirmed that the MLEC lines were *mycoplasma* negative.

### Proximity ligation assay

C20 human microglia cells were obtained from American Type Culture Collection and cultured in DMEM supplemented with 10% FBS and 1% penicillin/streptomycin. The cells were cultured in a controlled environment at 37 °C with 5% CO_2_. The experimental procedure involved seeding C20 cells on coverslips at 40% confluency and culturing the cells accordingly. Heparinase-treated cells were used as the background control and were prepared by digesting the cells for 1 h at 37 °C with heparinases I + III enzymes (R&D, Cat#7897-GH and Cat#6145-GH) at a concentration of 0.5 μg/ml (62). Subsequently, the cells were fixed with 4% paraformaldehyde in PBS for 10 min at RT and then blocked for 1 h using immunofluorescence blocking buffer (4% normal goat serum, 1% bovine serum albumin, 0.5% Triton in PBS) at 37 °C. Subsequently, cells were incubated overnight at 4 °C with primary antibodies (rabbit anti-human TREM2 from Cell Signaling Technology, Cat#91068, 1:300, and mouse anti-10E4 from AmsBio, 1:300) diluted in immunofluorescence blocking buffer. The next day, samples were washed thoroughly between steps, and incubated with 1:750 Thermo Fisher Scientific AlexaFluor secondary antibodies (anti-rabbit IgG 488 (Cat # A11008) and anti-mouse IgM 594 (Cat # A21044) for 1 h at 37 °C. After washing, samples were incubated with 1:10,000 4′,6-diamidino-2-phenylindole in PBS and mounted on slides using ProLong Gold Antifade mounting medium (Thermo Fisher Scientific, Cat# P36930). Imaging was conducted using a Leica SP8 confocal microscope with a 60× objective and 3× optical zoom, capturing images at a resolution of 1024 × 1024. Image analysis was conducted using ImageJ/Fiji (NIH) software (https://imagej.net/software/fiji/), and the Coloc2 plugin was used to determine the Pearson R correlation coefficient between TREM2 and HS immunofluorescence. We confirmed that the C20 cells were *mycoplasma* negative.

### Heparin, chemically modified heparin and heparin oligosaccharides

The porcine intestinal heparin (16 kDa) was from Celsus Laboratories. N-Des Hep (14 kDa) and 2-O-desulfated heparin (2-Des Hep, 13 kDa) were prepared based on Yates *et al.* (63). Additionally, 6-O-desulfated heparin (6-Des Hep, 13 kDa) was prepared in previous studies (19, 20). These chemically modified heparins were confirmed to have no anticoagulant activity *via* the amidolytic antifactor Xa assay and were negative for endotoxin using the Limulus test (19). Heparin oligosaccharides, including tetrasaccharide (dp4, Cat#H004), octasaccharide (dp8, Cat#H008), decasaccharide (dp10, Cat#H010), and hexadecasaccharide (dp16, Cat#H016), were from Iduron. These oligosaccharides were prepared by controlled partial depolymerization of unfractionated heparin by heparinase I followed by separation *via* high-resolution gel filtration.

### Assessing the interaction between TREM2 and heparin using surface plasmon resonance analysis

The SPR analysis was conducted using a Biacore 3000 instrument (Cytiva). The heparin chip used had been prepared in prior investigations by immobilizing biotinylated heparin on a sensor streptavidin SA chip from Cytiva (60, 62, 64, 65, 66). For the direct binding analysis, TREM2 (Sino Biological, Cat. # 11804-H08H) was appropriately diluted in a running buffer composed of 0.01 M Hepes, 0.15 M NaCl, 3 mM EDTA, and 0.005% surfactant P20, adjusted to pH 7.4 (referred to as HBS-EP buffer). TREM2 proteins at various concentrations were then injected over the heparin chip at a constant flow rate of 30 μl/min. After the injection phase, a buffer was passed over the sensor surface to facilitate dissociation. Following a 3-min interval, the sensor surface was regenerated by injecting 30 μl of 2 M NaCl solution. The response was continuously monitored over time (as a sensorgram) at 25 °C. To extract kinetic parameters such as the association rate constant (k_a_), dissociation rate constant (k_d_), and equilibrium dissociation constant (K_D_), the sensorgrams obtained from injections of various concentrations of TREM2 were globally fitted. This fitting procedure was performed using the BIAevaluation software version 4.0.1 (Cytiva, https://biaevaluation.software.informer.com/4.1/), assuming a 1:1 Langmuir model. The experiments were repeated three times. In the solution competitive binding analysis, the SPR experiment followed a similar protocol. wTREM2 at a concentration of 170 nM was premixed with 1000 nM of chemically modified heparins. Alternatively, TREM2 at a concentration of 250 nM was premixed with 1000 nM of heparin oligos, including dp4, dp8, dp10, and dp16, all in HBS-EP buffer.

### LMW HS microarray

A microarray chip was prepared by immobilizing ninety-six structurally defined LMW HS mimetic oligosaccharides at a concentration of 25 μM using established procedures (21, 67). Each compound was printed with 36 spots in a 6 × 6 square. The chip was then incubated with 100 μl His-tagged human TREM2 at a concentration of 10 μg/ml in PBS for 1 h at RT. Concurrently, 100 μl of 1 μM HIS Lite OG488-Tris NTA-Ni complex was also applied to the chip. Post incubation, nonspecifically bound fluorophores and proteins were washed off from the chip through two wash cycles. Fluorescence was excited at 488 nm using a GenePix 4300 scanner (Molecular Dynamics). The scanner's resolution was set at 5 μm precision. The resulting array images were analyzed using GenePix Pro 7.2.29.002 software (https://support.moleculardevices.com/s/article/GenePix-Pro-Software-Manual-Download-Page) to obtain mean fluorescence intensities *via* the software's array quality control feature. Automated detection localizes the printed spots; manual correction rectifies any discrepancies observed during this process. Finally, mean fluorescence intensities for each oligosaccharide spot were plotted against their respective identities using GraphPad Prism version 9.3.1 (https://www.graphpad.com/updates/prism-931-release-notes).

### RNA purification and quantitative RT-PCR

Total cellular RNA was extracted from C20 cells using TRIzol reagent (Invitrogen). Complementary DNA was synthesized using iScript cDNA synthesis kit (Cat #1708891, Bio-rad), according to the manufacturer’s instructions. Quantitative RT-PCR was carried out in 20-μl volumes that included 10 μl of iTaq Universal SYBR Green Supermix (Cat #1725124, Bio-Rad), 5 μl of diluted RT products as template, and 20 pmol of individual primer. Primer sequences used included the following: 5-ATGATGCGGGTCTCTACCAGTG-3 (forward) and 5-GCATCCTCGAAGCTCTCAGACT-3 (reverse) for human *Trem2*; 5-GCTCTTGTCTCGCCCTTTTGT-3 (forward) and 5- GTGGTGCAAGCCATTCCTAC-3 (reverse) for human *Ext1*; 5-ATGTGTGCGTCGGTCAAGTAT-3 (forward) and 5- AGAATGGGGCCAAAACTGAAA-3 (reverse) for human *Ext2*; 5-ACGCCCAGGAAGTTCTACTAC-3 (forward) and 5- GTTGTACGGGCAGTCCATGAA-3 (reverse) for human *Hs6st1*; 5- CATGGGGCCGAAAATCTTGGA-3 (reverse) for human *Hs6st2*; 5-TCCAGTGTCACGTTACCTGAG-3 (forward) and 5- TGTAGGTGCAATCCATAAACTCC-3 (reverse) for human *Hs6st3*; 5-GTCTCCTCTGACTTCAACAGCG-3 (forward) and 5- ACCACCCTGTTGCTGTAGCCAA -3 (reverse) for human *GAPDH*. All primers used in quantitative reverse transcription PCR were synthesized by Integrated DNA Technologies. The PCR amplifications (40 cycles at 95 °C for 5 s and at 60 °C for 30 s) were performed using the Bio-Rad CFX96 real-time system. For each target gene, the average Ct values were calculated from six replicates, which were then normalized to the average Ct values for GAPDH. These normalized values were used to calculate a value expressing the extent of knockdown relative to the nonspecific control siRNA, based on the 2^−ΔΔCT^ method.

### RNA interference transfection

C20 cells (2 ×10^5^/well) were plated in 6-well plates. Next day, RNA interference (RNAi) transfection was conducted using Dicer substrate siRNAs (DsiRNAs) purchased from Integrated DNA technologies or On-TARGET plus SMARTpool human *Hs6st3* from Horizon, and the Lipofectamine RNAiMAX transfection reagent (REF-13778; Invitrogen) following the manufacturer’s instructions, including NC-1 (IDT), a negative control DsiRNA with no homology to any human gene as a control. DsiRNA sequences used included the following: 5-GUCCUGAGUCUGGAUACUUUAGACA -3 for human *Ext1*; 5-CUACGAGAAGAAGUACUACUUCCCG-3 for human *Hs6st1*; 5-AACACCUGACAACUUCUGAAUAUUG-3 for human *Trem2*. Forty-eighthours after infection, the cells were harvested for RNA extraction or subjected to a function study. The transfection efficiency was monitored *via* quantitative reverse transcription PCR.

### ApoE internalization assay

The expression, purification, and conjugation of human ApoE3 with Alexa Fluor 488 (ApoE3-488) were previously described (23). C20 cells, including those transfected with either an empty vector or siRNA targeting *TREM2*, *Ext1*, *Hs6st1*, or *Hs6st3*, were seeded at a density of 3 × 10^4^ cells per well in 300 μl of DMEM supplemented with 10% FBS, 100 U/ml penicillin, and 100 μg/ml streptomycin. After overnight culture, the cells were washed twice with Dulbecco's phosphate-buffered saline and then incubated with 300 μl/well of DMEM containing ApoE3-488 (3 μg/ml) at 37 °C for 8 h. Subsequently, the cells underwent image analysis. They were covered with mounting medium containing 4′,6-diamidino-2-phenylindole and examined for internalized ApoE3-488 using a Keyence BZ-Z800 microscope. Image data were processed using ImageJ/Fiji (NIH) software (https://imagej.net/software/fiji/).

### Statistical analysis

Statistical analysis was carried out with Prism 8 for Macintosh. All data are presented as mean ± SD or mean ± SEM and analyzed using a student's *t* test for two-group comparison. *p* values less than 0.05 were chosen as a threshold for statistical significance.

## Data availability

The research data are available from the corresponding author.

## Supporting information

This article contains supporting information.

## Conflicts of interest

The authors declare that they have no conflicts of interest with the contents of this article.
