# Supplementary figures and images for: TREM2 on microglia cell surface binds to and forms functional binary complexes with heparan sulfate modified with 6-O-sulfation and iduronic acid
Source: J Biol Chem. 2024 Aug 17;300(9):107691. doi: 10.1016/j.jbc.2024.107691 (PMC11416269; doi:10.1016/j.jbc.2024.107691)

**
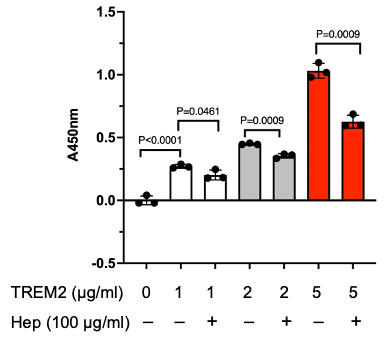
**

**Figure S1. Trem2 binding to wildtype *Ext1f/f*MLECs in the presence of heparin (100 g/ml).**

Supplement: Supplemental Figure S1 [file mmc1.docx]

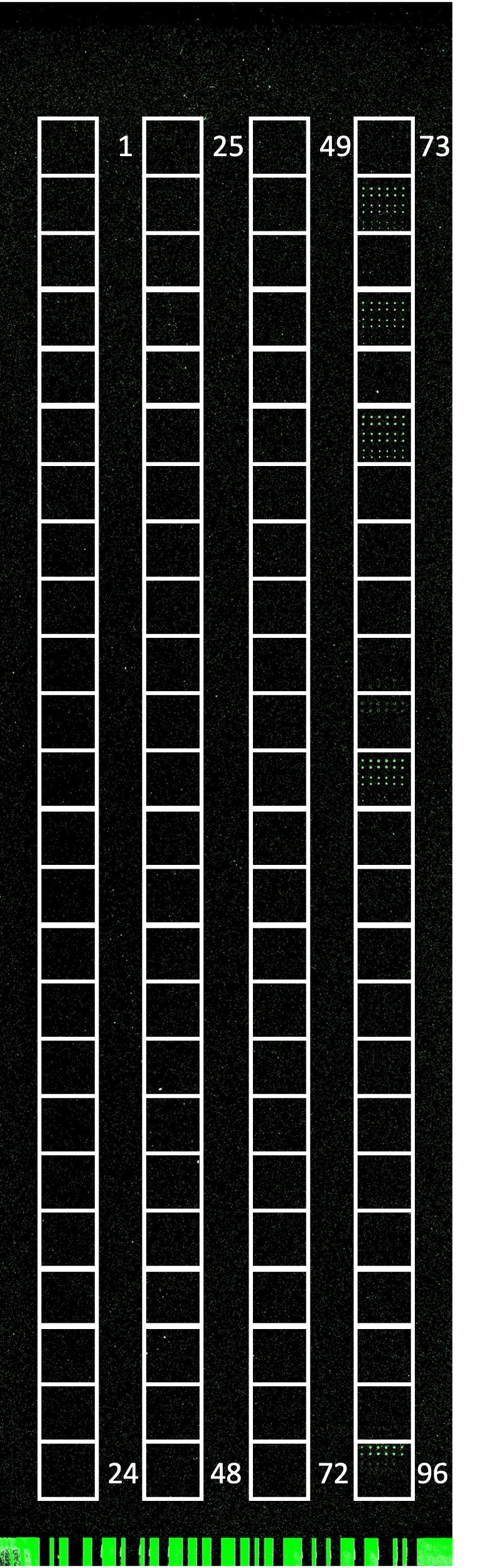


**Figure S4.** Raw fluorescent results of TREM2 binding LWHS microarray and Corresponding HS code.

Supplement: Supplemental Figure S4 [file mmc4.docx]

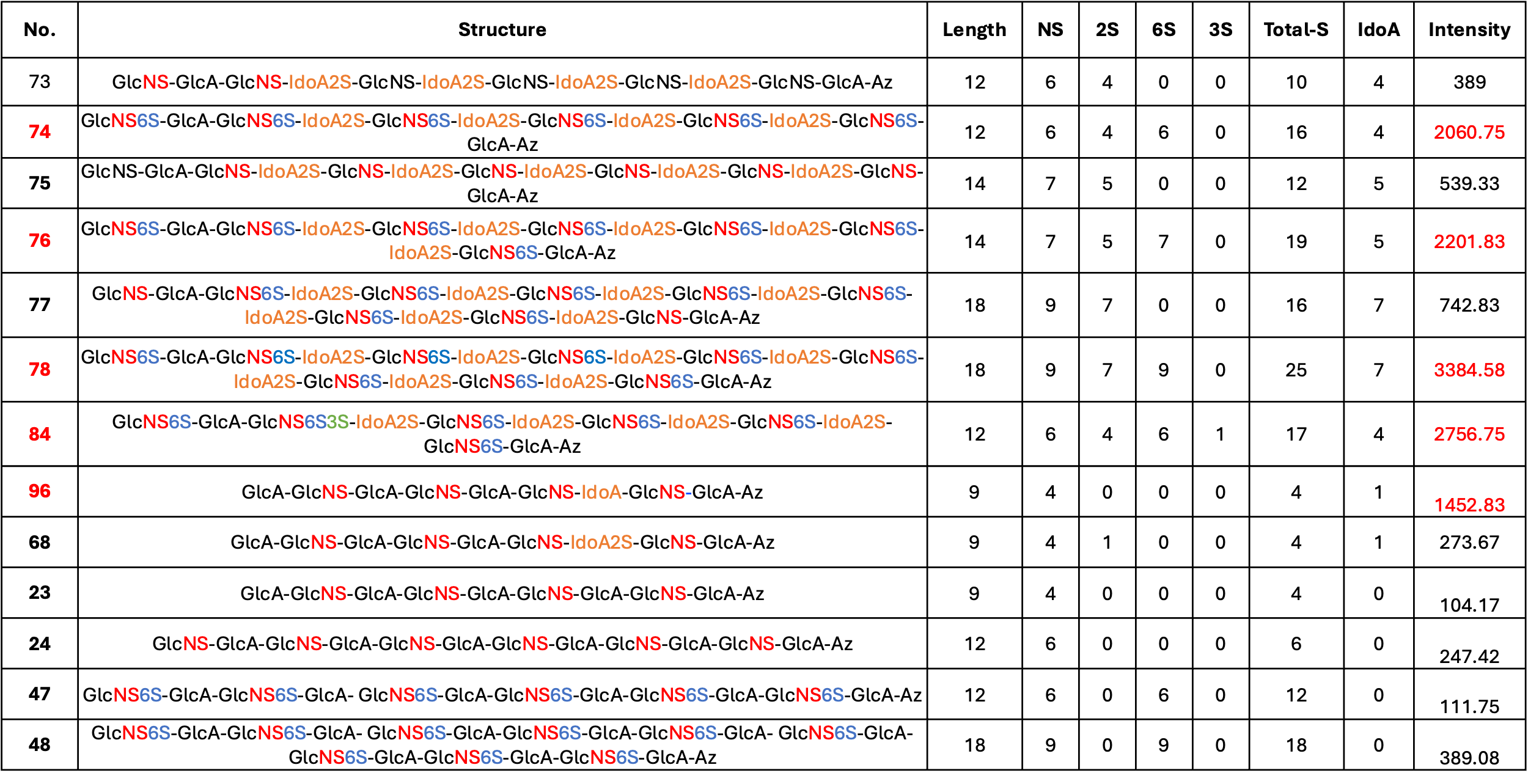


**Table S1.** LWHHS compounds` structure and their TREM2 binding intensities

Supplement: Supplemental Table S1 [file mmc6.docx]
