# Supplementary material for: TREM2 on microglia cell surface binds to and forms functional binary complexes with heparan sulfate modified with 6-O-sulfation and iduronic acid
Source: J Biol Chem. 2024 Aug 17;300(9):107691. doi: 10.1016/j.jbc.2024.107691 (PMC11416269; doi:10.1016/j.jbc.2024.107691)
Supplement: Supplemental Figure S2 [file mmc2.docx]

**Figure S2. Compound numbering (Cmpd #) and structures with symbol nomenclature for low molecular weight HS structures on the microarray.** Cmpds 1–96 are modified at the reducing end to contain an amine linker for adherence to the microarray chip.
