# Supplementary material for: TREM2 on microglia cell surface binds to and forms functional binary complexes with heparan sulfate modified with 6-O-sulfation and iduronic acid
Source: J Biol Chem. 2024 Aug 17;300(9):107691. doi: 10.1016/j.jbc.2024.107691 (PMC11416269; doi:10.1016/j.jbc.2024.107691)
Supplement: Supplemental Figure S3 [file mmc3.docx]

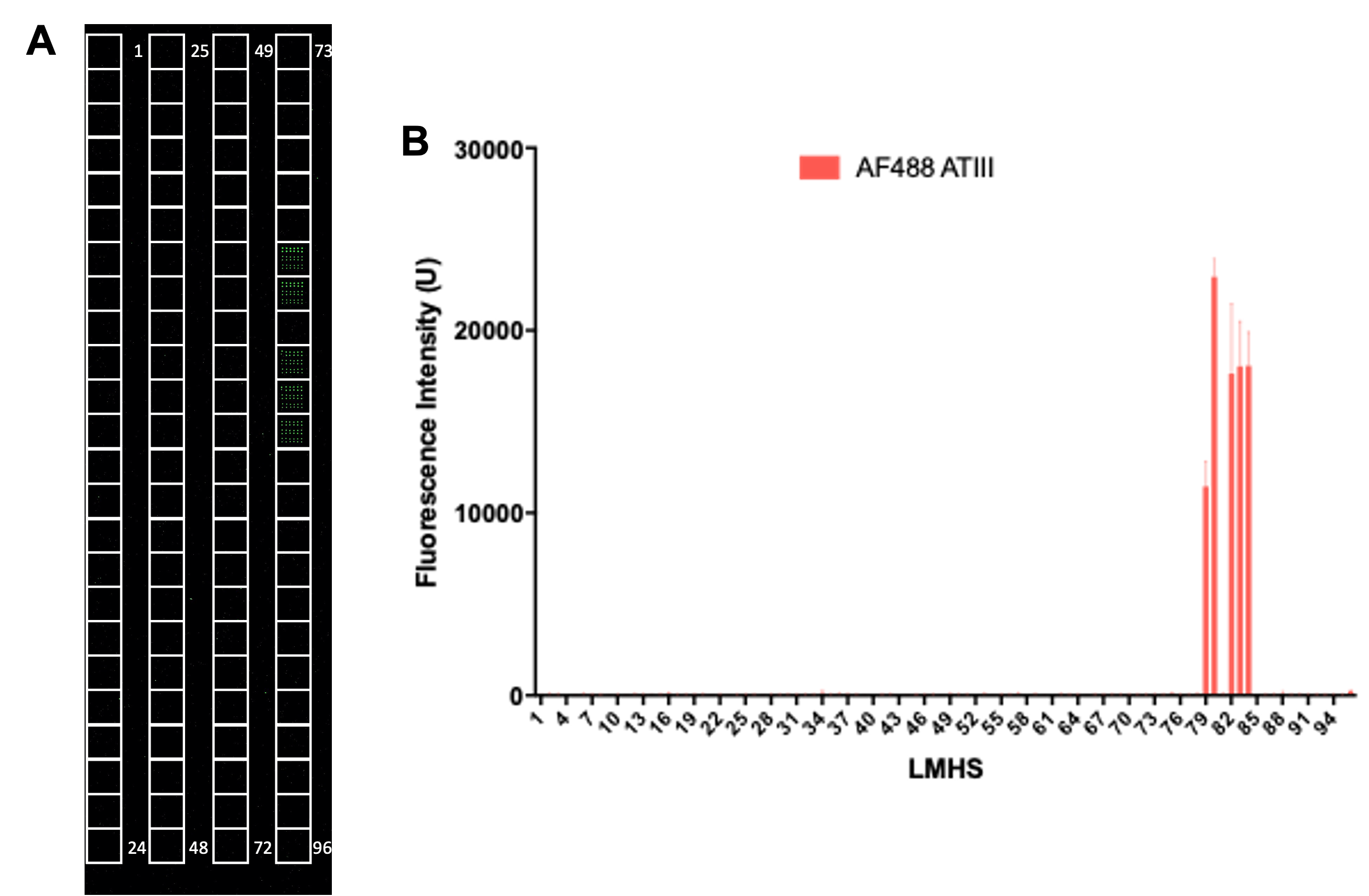


**Figure S3. Low molecular weight HS microarray of antithrombin III binding. (A)** Raw fluorescent results of the antithrombin III binding LMW HS microarray and corresponding HS code (numbered 1–96). (**B**) The complete results of antithrombin binding to an LMW HS microarray were visualized with OG488, and the 5 HS oligosaccharides known to bind antithrombin all showed positive binding in the assay.
