# Supplementary material for: TREM2 on microglia cell surface binds to and forms functional binary complexes with heparan sulfate modified with 6-O-sulfation and iduronic acid
Source: J Biol Chem. 2024 Aug 17;300(9):107691. doi: 10.1016/j.jbc.2024.107691 (PMC11416269; doi:10.1016/j.jbc.2024.107691)
Supplement: Supplemental Figure S5 [file mmc5.docx]

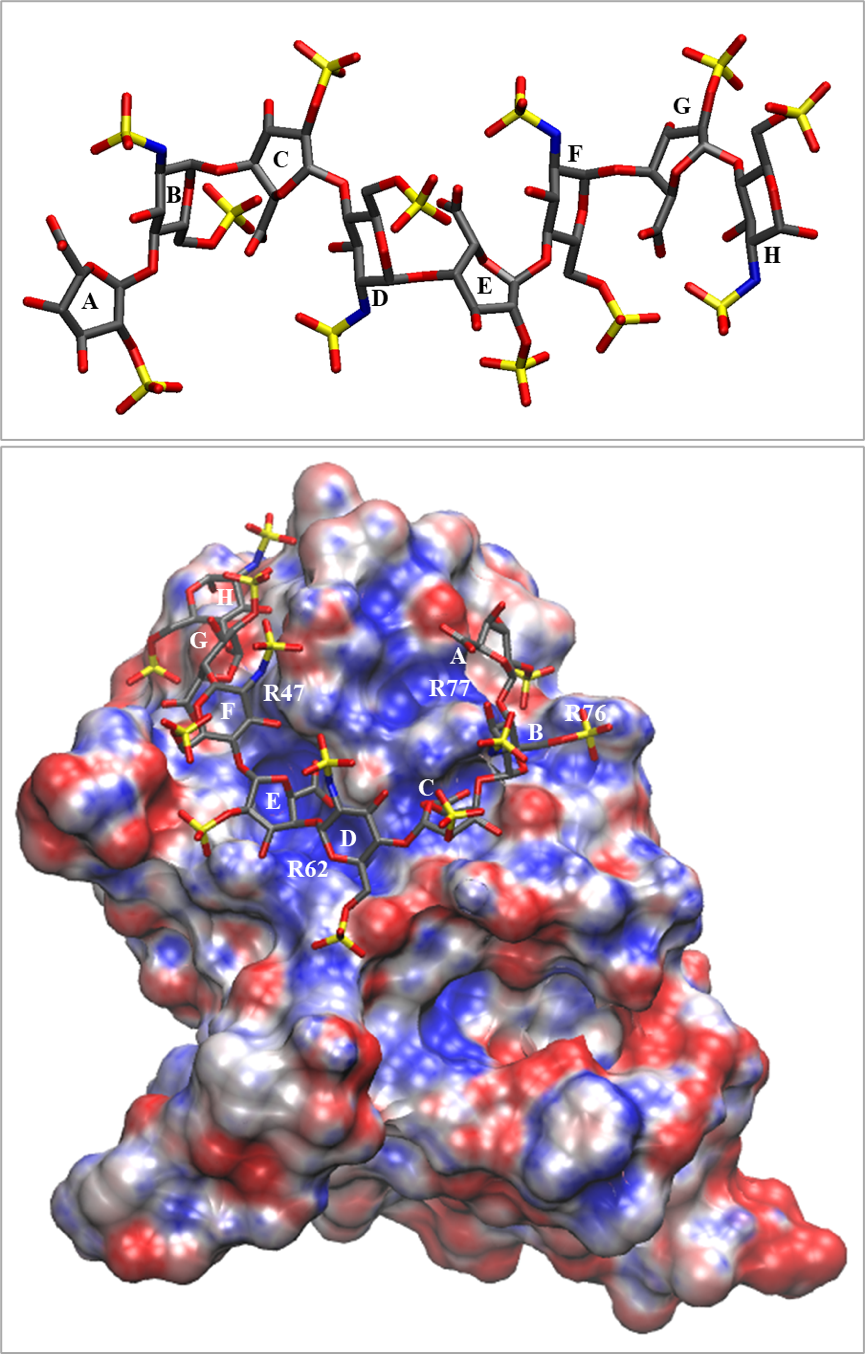


**Figure S5.** Upper: The ligand structure prior to docking is depicted as sticks, with colors corresponding to the atomic chemical elements (bottom figure). Lower: The structure of heparin octasaccharide used for docking (top figure). Top docked pose for the heparin fragment with TREM2. The protein structure is shown as a solvent accessible surface colored according to the electrostatic potential from red to blue indicating electrostatically negative to positive.
