# Supplementary material for: TREM2 on microglia cell surface binds to and forms functional binary complexes with heparan sulfate modified with 6-O-sulfation and iduronic acid
Source: J Biol Chem. 2024 Aug 17;300(9):107691. doi: 10.1016/j.jbc.2024.107691 (PMC11416269; doi:10.1016/j.jbc.2024.107691)
Supplement: Supplemental Tables S2 and S3 [file mmc7.docx]

| **Octa-saccharide residue** | **Interactions** |
| --- | --- |
| A | O2-SO_3_---R76, O2-SO_3_---R77 |
| B | O6-SO_3_---R76 |
| C | COO---S65, COO…R77 |
| D | O6-SO_3_---R62 |
| E | COO---S65, COO…R47, O2-SO_3_---R62 |
| F | N-SO_3_---R47, N-SO_3_---N68 |
| G | O2-SO_3_---N68 |
| H | N-SO_3_---W70 |

**Table S3.** Glycosidic ɸ/ψ-torsion angles^a,b^ before and after docking.

| **Octa-saccharide Linkage** | **Before** | **After** |
| --- | --- | --- |
| A-B | -51.3 / 121.4 | -85.0 / 111.2 |
| B-C | 67.8 / 110.8 | 96.9 / 88.9 |
| C-D | -51.3 / 121.4 | -106.4 / 53.9 |
| D-E | 60.4 / 104.1 | 97.4 / 15.1 |
| E-F | -49.1 / 127.1 | -98.9 / 102.0 |
| F-G | 62.4 / 107.2 | 109.1 / 79.9 |
| G-H | -50.6 / 123.3 | 139.7 / 107.5 |

**Table S2.** Protein-ligand interactions between charged moieties in the TREM2-heparin complex. Hydrogen bonds are shown as (---) and salt bridges as (…). ^a^Values in degrees. ^b^ ɸ:O5-C1-O(x)-C(x), ψ: C1-O(x)-C(x)-C(x-1)
