# Supplementary material for: TREM2 on microglia cell surface binds to and forms functional binary complexes with heparan sulfate modified with 6-O-sulfation and iduronic acid
Source: J Biol Chem. 2024 Aug 17;300(9):107691. doi: 10.1016/j.jbc.2024.107691 (PMC11416269; doi:10.1016/j.jbc.2024.107691)
Supplement: Supporting Information [file mmc8.docx]

**TREM2 on microglia cell surface binds to and forms functional binary complexes with heparan sulfate modified with 6-O-sulfation and iduronic acid**

**Running title: TREM2 binds to specifically structured heparan sulfate**

Ilayda Ozsan McMillan^1^, Li Liang^2^, Guowei Su^3^, Xuehong Song^1^, Kelly Drago^1^, Hua Yang^1^, Claudia Alvarez^1^, Amika Sood^4^, James Gibson^2^, Robert Woods^4^, Chunyu Wang^2^, Jian Liu^5^, Fuming Zhang^2^, Tom J. Brett^6^, Lianchun Wang^1^

^1^ Department of Molecular Pharmacology and Physiology, University of South Florida Morsani College of Medicine, Tampa, FL, USA.

^2^ Center for Biotechnology and Interdisciplinary Studies, Departments of Chemistry and Chemical Biology, Department of Biological Sciences, Rensselaer Polytechnic Institute, Troy, NY, USA.

^3^ Glycan Therapeutics, Raleigh, NC 27606, USA.

^4^ Complex Carbohydrate Research Center, University of Georgia, Athens, Georgia, 30602, USA.

^5^ Division of Chemical Biology and Medicinal Chemistry, University of North Carolina at Chapel Hill, Eshelman School of Pharmacy, Chapel Hill, NC 27599, USA.

^6^ Division of Pulmonary and Critical Care Medicine, Department of Internal Medicine, Washington University School of Medicine, St. Louis, USA

Corresponding author: Lianchun Wang, MD

E-mail: [lianchunw@usf.edu](mailto:lianchunw@usf.edu)

**Keywords: TREM2, heparan sulfate, binary complex, structure-function, microglia**

**List of the materials**

1. Method
2. Results
3. Figure S1-5
4. Table S1-2

**Method**

**In silico modeling of the TREM2-HS interaction**. To create a model of the interaction between heparin and TREM2, the 3D structure of a heparin octa-saccharide fragment ([IdoA2S-GlcNS6S]_4_) with all IdoA2S sugars in ^2^S_O_ conformation was generated using GLYCAM-Web server (<https://glycam.org/>). The A chain from a crystal structure of TREM2 (PDB ID 5ELI) was downloaded from the protein databank (https://www.rcsb.org/) and used as the receptor. Input files were prepared using AutoDock Tools 1.5.6 (ADT) (1). Docking was performed with Vina-Carb (2), a version of AutoDock Vina (3) derived for use with carbohydrates (4). A docking grid box (24Å, 32Å and 24Å in the X, Y, and Z axes, respectively) was placed at the center of mass of arginine residues 46, 47, 76 and 77 as they form the most electrostatically positive patch on the surface of the protein. During docking, an exhaustiveness value of 80 was applied, and all exocyclic torsion angles in the ligand were treated as flexible. The Visual Molecular Dynamics (VMD) program (5) was employed for image rendering. Interactions between the protein and ligand were detected using the Protein-Ligand Interaction Profiler (PLIP) webservice (6).

**Results**

**In silico modeling of the TREM2-HS interaction.** The focused docking resulted in ligand 20 poses. The top ranked pose (Figure S5), determined by theoretical interaction energy, was selected for visualization and further analysis. As identified by PLIP, the heparin fragment formed hydrogen bonds and/or salt bridge interactions with seven residues of the protein: four arginines (R47, R62, R76, and R77), one asparagine (N68), one serine (S65), and one tryptophan (W70) (Table S2). The values of the glycosidic torsion angles (Table S2) were all within allowable regions of the potential energy surface (6, 7). The modeling indicate that an octamer-fragment of heparin could occupy the entire putative binding site in TREM2. The experimental observation that binding to immobilized heparin fragments increases with heparin chain length may arise from mass transfer (or ligand rebinding) effects due to the increase in the number of binding epitopes or an alternative binding mode. Further modeling and/or experimental studies are needed to quantify the roles of each sulfate moiety and related amino acid residues; however, the present model provides a basis for designing such studies.

**Reference**

1. Morris, G. M., Huey, R., Lindstrom, W., Sanner, M. F., Belew, R. K., Goodsell, D. S. *et al.* (2009) AutoDock4 and AutoDockTools4: Automated docking with selective receptor flexibility J Comput Chem **30**, 2785-2791 10.1002/jcc.21256

2. Nivedha, A. K., Thieker, D. F., Makeneni, S., Hu, H., andWoods, R. J. (2016) Vina-Carb: Improving Glycosidic Angles during Carbohydrate Docking J Chem Theory Comput **12**, 892-901 10.1021/acs.jctc.5b00834

3. Trott, O., andOlson, A. J. (2010) AutoDock Vina: improving the speed and accuracy of docking with a new scoring function, efficient optimization, and multithreading J Comput Chem **31**, 455-461 10.1002/jcc.21334

4. Trott, O., andOlson, A. J. (2010) AutoDock Vina: Improving the speed and accuracy of docking with a new scoring function, efficient optimization, and multithreading Journal of Computational Chemistry **31**, 455-461 10.1002/jcc.21334

5. Humphrey, W., Dalke, A., andSchulten, K. (1996) VMD: visual molecular dynamics J Mol Graph **14**, 33-38, 27-38 10.1016/0263-7855(96)00018-5

6. Salentin, S., Schreiber, S., Haupt, V. J., Adasme, M. F., andSchroeder, M. (2015) PLIP: fully automated protein-ligand interaction profiler Nucleic Acids Res **43**, W443-447 10.1093/nar/gkv315

7. Nivedha, A. K., Makeneni, S., Foley, B. L., Tessier, M. B., andWoods, R. J. (2014) Importance of ligand conformational energies in carbohydrate docking: Sorting the wheat from the chaff J Comput Chem **35**, 526-539 10.1002/jcc.23517
